# Identification of acute kidney injury in African children with severe malaria: a multinational individual participant data meta-analysis

**DOI:** 10.21203/rs.3.rs-8106913/v1

**Published:** 2025-11-23

**Authors:** Caitlin Bond, Anthony Batte, Folake Afolayan, Nicholas Anstey, Quique Bassat, Philip Bejon, James A. Berkley, Rhys D. R. Evans, Stuart L. Goldstein, Michael T. Hawkes, Olayinka Ibrahim, Peace Imani, Chandy C. John, Kevin C. Kain, Claire Liepmann, Kevin Marsh, Ruth Namazzi, Margaret Nakuya, Nicole O’Brien, Lere P Oluwadare, Robert O. Opoka, Jonathan Sserunkuuma, Hunter Wynkoop, Andrea L. Conroy, maKID Consortium

**Affiliations:** Indiana University School of Medicine; Makerere University College of Health Sciences; SUNY Upstate Medical University; Charles Darwin University; Universitat de Barcelona; University of Oxford; University of Oxford; University College London; Cincinnati Children’s Hospital Medical Center; University of British Columbia; University of Global Health Equity; Baylor College of Medicine; Indiana University School of Medicine; University of Toronto; Indiana University School of Medicine; University of Oxford; Makerere University College of Health Sciences; Makerere University College of Health Sciences; The Ohio State University; University College Hospital, Ibadan; Aga Khan University Medical College Africa; Mbarara University of Science and Technology; The Ohio State University; Indiana University School of Medicine

**Keywords:** acute kidney injury, severe malaria, mortality, KDIGO, meta-analysis, Africa

## Abstract

**Background.:**

Historically, acute kidney injury (AKI) has been an underappreciated complication in children with severe malaria. We conducted an individual patient data meta-analysis to model the impact of AKI and its complications on inpatient mortality in African children hospitalized with malaria.

**Methods.:**

Studies were identified using MEDLINE, EMBASE, Scopus, and PubMed with no language restrictions, as well as through outreach at scientific meetings. Investigators were contacted about participation in the study. Eligible studies included African children hospitalized with severe *Plasmodium falciparum* malaria, a serum creatinine measurement, and mortality assessed. The primary exposure was AKI, defined using Kidney Disease Improving Global Outcomes (KDIGO) criteria based on serum creatinine. The primary outcome was all-cause in-hospital mortality, and dialysis requirement was a secondary outcome. Data were standardized and cleaned, and random-effects meta-analyses were conducted to generate pooled estimates.

**Results.:**

We included 18 studies involving 8 countries and 13,528 children aged 3 months to 16 years over a 33-year period with 951 deaths (7%). The frequency of KDIGO-defined AKI was 47% (95% confidence interval: 46% to 48%), with 46% of children having KDIGO-defined Stage 1 AKI, 30% having Stage 2, and 23% having Stage 3 AKI. Overall, KDIGO-defined AKI was associated with a 2.87 increased odds of mortality (95% CI 2.28 to 3.61) with a monotonic increase in mortality across AKI stage. AKI was a robust risk factor for mortality across era, sex, region, and subgroups. The KDIGO AKI definition was operationalized using simplified age-based thresholds to support bedside recognition of AKI with comparable mortality performance. The WHO-defined AKI threshold (creatinine value > 3mg/dL) occurred in 3.4% (238) of children. All 238 cases identified by WHO criteria were classified as having AKI using KDIGO and age-based definitions, but WHO missed 93% (3059/3297) of additional AKI cases.

**Discussion.:**

AKI was strongly associated with mortality in African children with severe malaria. Creatinine thresholds can be operationalized to facilitate early AKI recognition. These findings have clinical implications for the implementation of kidney-protective care and for enhanced monitoring of children with severe malaria.

**Other.:**

Funded by National Institutes of Health (1R01AI165946), registered number PROSPERO (CRD42024560012).

## Introduction

Malaria remains an important global cause of morbidity and mortality in children, with 263 million cases and 597,000 associated deaths in 2023 [1]. Africa contributes to a disproportionate burden of clinical malaria cases, accounting for 94% of malaria cases and 95% of malaria deaths [1]. Severe malaria is a life-threatening condition characterized by multiple organ dysfunction [2]. Acute kidney injury (AKI) is defined as an abrupt loss of kidney excretory function over hours to days, diagnosed based on an increase in serum creatinine or a decrease in urine output [3]. An estimated 25% of pediatric hospitalizations are complicated by AKI globally [4]. AKI-associated mortality is highest in low-income settings where access to specialized nephrology care and dialysis remains limited [5]. In Africa, severe malaria is a leading cause of AKI as well as a driver of acute dialysis needs among pediatric patients [6]. Mortality associated with AKI remains unacceptably high due to delays in recognition and early management [7].

The nephrology community has developed consensus guidelines to standardize AKI recognition (e.g., the Kidney Disease: Improving Global Outcomes, KDIGO); however, these criteria have not been adopted in the definition of severe malaria in the current WHO treatment guidelines [2]. New guidelines and strategies are urgently needed to facilitate the early recognition of AKI in severe malaria to improve clinical management and mitigate the risks of AKI progression [8]. The KDIGO guidelines represent evidence-based criteria to define, classify, and stage clinically meaningful increases in serum creatinine that are associated with mortality across diverse patient populations, and support a rational age-appropriate definition of AKI [9].

In this individual-participant data meta-analysis, we evaluate the association between AKI and mortality (primary analysis). We also describe the frequency of AKI across multiple pediatric malaria cohorts, varying by geography and time. We compare AKI definitions (KDIGO and WHO) and operationalize an AKI definition to support AKI recognition in children. Finally, we examine AKI-related complications (hyperkalemia, elevated BUN) and their association with dialysis and mortality.

## Methods

### Study design

We followed the Preferred Reporting Items for a Systematic Review and Meta-analysis Individual Participant Data (PRISMA-IPD) guidelines [10].

The primary exposure in this study was AKI defined by KDIGO criteria [11]. The primary outcome was in-hospital mortality. Criteria for study inclusion of individual patients in the meta-analysis required all the following: (1) age 3 months to < 18 years of age; (2) hospitalized with *Plasmodium falciparum* infection using blood smears or rapid diagnostic tests; (3) at least one serum creatinine measurement; and (4) hospital outcome (survival vs. non-survival) was recorded. Secondary exposures included AKI-related complications of hyperkalemia (potassium > 5.5 mmol/L) and elevated blood urea nitrogen (BUN, > 20mg/dL). Subgroup analyses were conducted by biological sex, age group, nutritional status (severe wasting), decade, and region of enrollment. Severe wasting was defined as a weight-for-height z-score < − 3 standard deviations based on WHO anthropometric age and sex-based z-scores. AKI frequency estimates were restricted to studies where creatinine was measured using an isotype dilution mass-spectrometry (IDMS)-traceable assay. Study quality and risk of bias were evaluated using the Newcastle-Ottawa Scale [12] assessing study quality across three domains: 1) Selection- 6 points (representativeness of the exposed cohort, selection of the non-exposed cohort, ascertainment of exposure, demonstration that outcome of interest was not present at the beginning of the study); 2) Comparability- 2 points; 3) Outcome- 5 points (assessment of outcome, follow-up was long enough for outcomes to occur, adequacy of follow-up of cohorts) [12]. Risk of bias by study is presented in Additional File 1, Table S1.

### Search strategy

To identify studies for inclusion, a literature search was conducted using MEDLINE, EMBASE, Scopus, and PubMed with no language restrictions. An initial pilot was conducted to ensure the search strategy identified known articles of interest. The search included three concepts using MESH terms and keywords (MESH: creatinine) AND (MESH: malaria, falciparum; keywords: severe malaria, falciparum malaria, complicated malaria) AND (MESH: child; keywords: pediatric, infant, adolescent). The initial search was conducted on February 25th, 2022, and was repeated on February 25th, 2025. Results were extracted to Covidence for further eligibility assessment by authors CDL and ALC. Additional searches to identify studies included a search of the grey literature, a review of reference lists of published work, outreach through social media, outreach to investigators in the fields of pediatric nephrology and global health through email and conference presentations, and a review of published cohorts of children with severe malaria to determine if appropriate data were collected.

Studies were eligible for inclusion if they were observational or randomized controlled trials and individual participant data were provided. Published and unpublished data were included. Studies that included a broader population of hospitalized children were eligible; however, children without malaria were excluded. Data collected using whole blood point-of-care creatinine tests were excluded due to issues of creatinine overestimation. The details of each study are provided in the supplemental methods (Additional File 2).

### Data management

Deidentified data for each study were reviewed and cleaned to create a standard set of variables, including a study identifier, participant identifier, age, height, weight, parasite density, and additional laboratory variables (hemoglobin, potassium, blood urea nitrogen) [13–21]. Outcomes included death (primary) and dialysis (secondary). Each dataset was reviewed, and the data were standardized and cleaned to remove participants who were less than three months or greater than 18 years old, or those with missing creatinine or mortality information.

### Defining acute kidney injury

AKI was defined using the 2012 KDIGO criteria [11] based on fold change in admission creatinine from baseline. Stage 1 AKI was a 1.5 to < 2.0-fold increase in creatinine, stage 2 AKI was a 2.0 to < 3.0-fold increase, stage 3 AKI was ≥ 3.0-fold increase in creatinine. Baseline creatinine was imputed assuming a normal GFR of 120mL/min per 1.73m^2^ and using age-based equations to back-calculate baseline creatinine, as has been validated in the pediatric literature [22]. Children with a creatinine value less than 0.4mg/dL were not considered to have AKI, irrespective of the fold change in creatinine. Sensitivity analyses evaluating AKI by creatinine threshold and age category are presented in Additional File 1, Figure S1. Renal impairment was defined as a creatinine > 3mg/dL (265 umol/L) based on WHO 2014 criteria [2]. AKI was operationalized using simplified age-based creatinine thresholds derived from rounded and batched estimates of baseline creatinine (Table 1) multiplied by the fold-change in creatinine for each stage.

### AKI frequency

We summarized AKI frequency descriptively by study and by calendar year. Within each study, we calculated the proportion meeting KDIGO AKI with binomial (Wald) 95% CIs and displayed an overall reference line equal to the pooled proportion (Σ cases / Σ N). To visualize temporal patterns, we aggregated by year and fit LOESS smoothed weights by annual sample size. Because creatinine assay practices changed over time, we restricted this analysis to data collected from 2008 onwards and, separately, to low-risk-of-bias studies shown in Additional File 1.

### Meta-analysis

Individual participant data (IPD) from 18 studies across nine countries were analyzed using a two-stage approach as the primary approach. One study from Kenya was split into two time periods due to changes in creatinine assays over time [23]. Within each study, we estimated the association between AKI and in-hospital mortality with Firth logistic regression to address sparse cells and separation [24], adjusting for age in all studies and assay type only in studies that used > 1 creatinine method. Study-specific log odds ratios (ORs) were pooled using a random-effects model with restricted maximum likelihood estimation and Hartung–Knapp confidence intervals [25]. Heterogeneity was summarized with Cochran’s Q and I^2^, and a prediction interval shown in forest plots. Sensitivity analysis included: (i) repeating the two-stage analysis with conventional logistic regression, (ii) repeating both approaches to low risk-of-bias studies, and (iii) a one-stage mixed-effects logistic model with a random intercept for study (fixed AKI effect) adjusting for age and assay type (when applicable).

#### Subgroups.

We fit separate mixed-effect logistic models (random intercept for study) for each subgrouping variable (age, decade, region, sex, assay method, severe wasting, severe malaria) including an AKI × subgroup interaction. We report contrasts (the adjusted odds ratio (aOR) for AKI) within each subgroup and show the overall AKI effect as a reference line in the plots.

#### Secondary exposures.

For secondary exposures, we evaluated hyperkalemia and elevated BUN using the same subgroup framework, adjusted for age. Because between-study heterogeneity was larger for these exposures, corresponding two-stage meta-analyses are presented in Additional File 1.

#### Covariate strategy.

We adjusted only for variables with meaningful within-study variation and a confounding role. Age was included in all models; assay type was included only in the one study with > 1 method. Variables that were constant within studies (e.g., region, decade, sex) or likely to be on the causal pathway (e.g., antimalarial regimen) were not included in the primary adjustment set, but were explored in sub-group analysis.

### Mediation

To quantify the extent to which the AKI–mortality association operates through hyperkalemia or elevated BUN, we used logistic mediator and outcome models that included study fixed effects and study-clustered inference. Models were adjusted for age, sex, decade, and region. If fixed effects were not estimable in sparse strata, we refit without them but retained clustering. We estimated average causal mediation effects, average direct effects, total effects, and proportion mediated using quasi-Bayesian Monte Carlo with 2,000 simulations.

All analyses were performed using R version 4.3.2 and Stata version 18.

### Data Access and Availability

Source data can be found at the following link. https://doi.org/10.7910/DVN/EBQNQD (Harvard Dataverse) [26].

## Results

**Figure 1:**
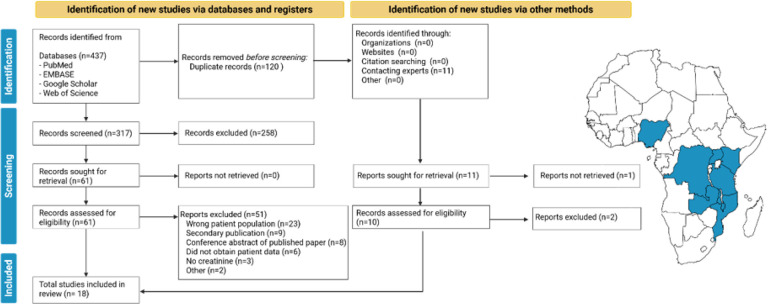
Flow chart of search strategy and map depicting countries represented.

13,528 children, aged between 3 months and 16 years, from 8 countries spanning 33 years were included in the study (Figure 1). While participant eligibility extended to 18 years, no participants older than 16 years were recruited across the studies. Most participants were from East Africa (89%) and 58% were from Kilifi, Kenya. Study characteristics are presented in Table 2.

### AKI frequency

**Figure 2:**
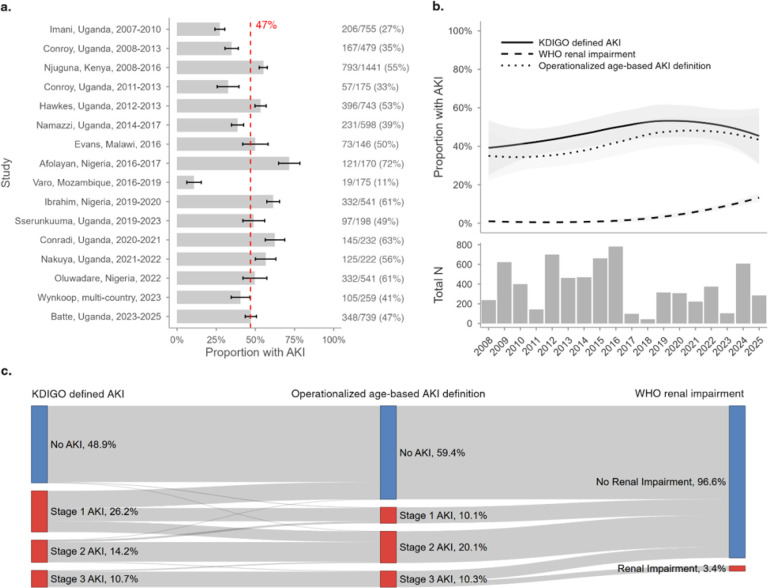
Acute kidney injury (AKI) frequency across studies and over time. a) Proportion of children with AKI across included studies after 2007, using KDIGO criteria. Bars represent point estimates of AKI frequency, and black error bars indicate 95% confidence intervals based on binomial standard errors. Total case numbers and sample sizes are displayed on the right. The red dashed line indicates the overall pooled AKI frequency. b) Smoothed temporal trend in AKI frequency from 2008 to 2025. The top panel shows LOESS curves (black lines), according to AKI definition, fit to annual AKI proportions, with shaded gray ribbons indicating 95% confidence interval. The bottom panel shows total sample sizes with available creatinine data for each year. c) Flow diagram showing classification concordance between three AKI definitions. KDIGO-defined AKI stages (left) are mapped to the operationalized age-based definition (center) and WHO renal impairment criteria (right). Colored bars indicate AKI presence according to definition, with percentages showing the proportion of the total cohort in each category. *The Sserunkuuma, Uganda (2019–2023) study is not shown in panel b, since year of enrollment was not recorded for individual participants (only overall totals were available). See Supplemental Figure S2 for a version of this figure only including studies with low risk-of-bias*.

To assess the frequency of AKI over time, data from 2008 onward were used, following the implementation of isotope dilution mass spectrometry (IDMS)-traceable assays. Overall, 3,297 children had AKI (47% [95% CI 46% to 48%]) (Figure 2) with no difference in AKI frequency over time. Of the children with AKI, 46% had KDIGO-defined Stage 1 AKI, 30% had Stage 2, and 23% had Stage 3 and 3.4% had WHO-defined renal impairment.

KDIGO-AKI frequency varied by age and was lowest in older children (under 2 years, 45%; 2–4 years, 50%; 5–9 years, 47%; 10 years or older, 38%; p<0.0001). When comparing KDIGO-defined AKI and WHO-defined renal impairment, 93% (3059/3297) of KDIGO-defined AKI cases were missed when using a threshold of 3mg/dL. When stratified by KDIGO-defined AKI stage, all WHO-defined AKI cases fell within Stage 3; yet the WHO definition still missed 63% of Stage 3 cases. AKI frequency using KDIGO definitions and minimum creatinine thresholds, as well as the WHO creatinine threshold, was modeled by age category and calendar time (Additional File 1, Figure S1). With increasing creatinine thresholds, the frequency of AKI declined and had the largest impact on children <2 years of age.

### Mortality

The potential association between KDIGO-defined AKI and in-hospital mortality was examined across all studies, including data collected before the implementation of IDMS-traceable assays. There were 951 deaths across all studies (7.0%). The mortality rates were 9.1% among patients with AKI and 3.5% in patients without AKI, respectively. There was a decline in malaria mortality over time, with a mortality rate of 13.1% before 2000, decreasing to 7.5% between 2000 and 2010, 5.0% between 2010 and 2020, and 4.6% after 2020. Mortality rates were consistent across all age groups and did not differ by sex (7.0% in females vs. 7.1% in males). In-hospital mortality was 8.4% and 4.8% prior to and after 2011, respectively, when artemisinin-derivatives were recommended as the first-line therapy for African children with severe malaria.

### Association between AKI and mortality

**Figure 3:**
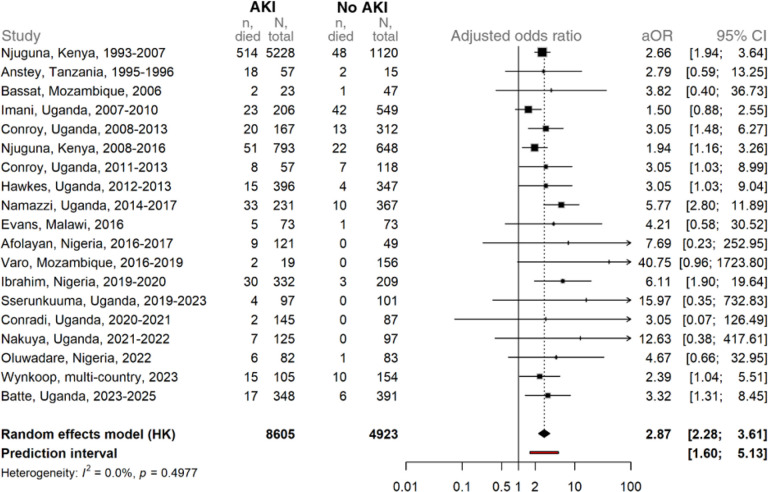
Association between acute kidney injury (AKI) and mortality in African children with severe malaria. Forest plot of age- and assay-adjusted odds ratios (aOR) for mortality associated with KDIGO-defined AKI, estimated using Firth logistic regression. Each square represents the study-specific effect estimate; horizontal lines indicate 95% confidence intervals. Square size is proportional to the study’s weight in the meta-analysis. The diamond shows the pooled random-effects estimate with its 95% confidence interval, while the red bar indicates the prediction interval (the expected range of effects in future similar studies). Numbers in the table denote, for each study: deaths and totals among participants with AKI, and deaths and totals among participants without AKI. Sensitivity analysis shown in Additional File 1, Supplemental Figures S2 and S3. *Njuguna, Kenya, was separated into two study periods (1993–2007 and 2008–2016). Adjustment for assay type was applied only in studies where more than one method was used*.

AKI was associated with a pooled odds ratio of in-hospital mortality of 2.87 (95% CI 2.28 to 3.61) across all studies (Figure 3), adjusting for age and assay method using a two-stage approach. There was a monotonic increase in mortality with increased AKI stages, with pooled mortality of 4.8%, 6.7% and 15% for stages 1 to 3, respectively (Table 2) compared to 3.5% in children without AKI. The results were comparable when calculating the fold change in creatinine from estimated baseline and simplified age-based thresholds (adjusted odds ratio 2.81 [95% CI 2.23, 3.54], Additional File 1, Figure S5). In a sensitivity analysis evaluating KDIGO-defined AKI on mortality, excluding all children who met the WHO definition of renal impairment, AKI remained a significant predictor of mortality with an odds ratio of 2.65 (95% CI 2.12 to 3.33; Additional File 1, Figure S6).

**Figure 4:**
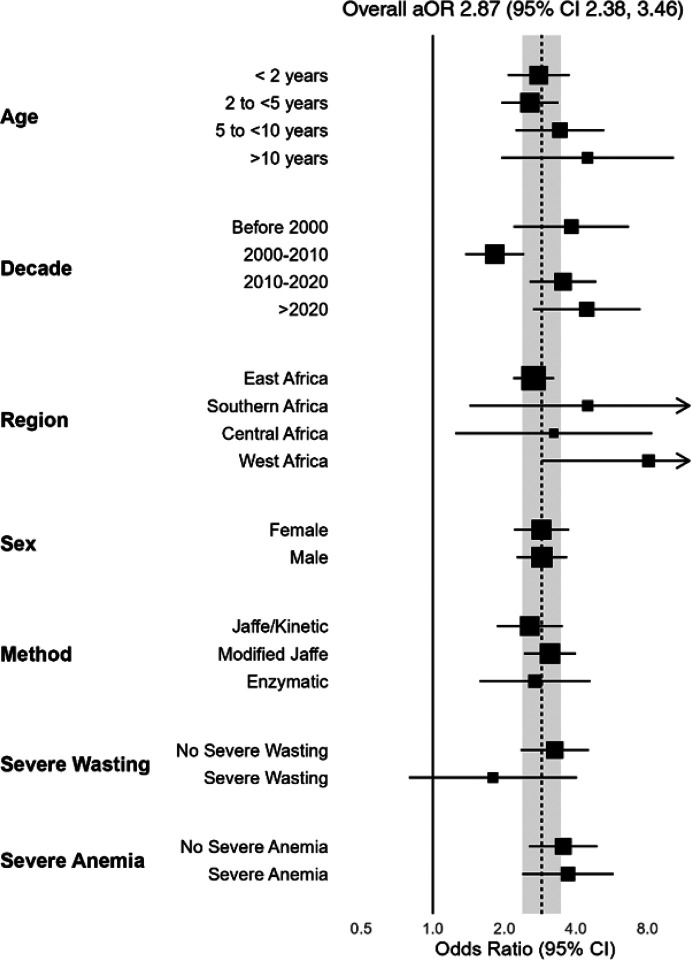
Relationship between AKI and mortality across sub-groups. A separate mixed effect logistic regression model was fit for each sub-group, adjusting for age and assay type if appropriate. The dashed line and shaded are represent the overall effect of AKI on mortality (adjusted odds ratio [aOR] and 95% confidence interval [CI], adjusted for age and assay, using a one-stage approach. Values shown in Additional File 1, Table S2.

As a sensitivity analysis, a one-stage approach was also used, which yielded a similar adjusted odds ratio (2.87 [95% CI 2.38 to 3.46], Figure 4) adjusting for age and assay method. The relationship between AKI and mortality was maintained across all pre-specified subgroups (Figure 4).

### Secondary outcome: dialysis

Among 13,528 participants, 78 received acute dialysis (0.6%) in Uganda and Nigeria. Dialysis was first administered in 2014, with a gradual increase in access over time, with 4.7% of children with severe malaria receiving dialysis in 2024 to 2025. In studies where dialysis was available, 45/1640 (2.7%) received hemodialysis, while 11/1640 (0.7%) received peritoneal dialysis. Mortality was 18.0% in children who received dialysis and was higher among children who received peritoneal dialysis (54.6%) compared to hemodialysis (11.1%). Using mixed-effects logistic regression with a random intercept for study, mortality was 62% lower in settings with dialysis available (OR, 0.38; 95% CI, 0.25 to 0.59), adjusting for child age, sex, AKI, hyperkalemia, and uremia.

### Secondary exposures: hyperkalemia and elevated BUN

**Figure 5:**
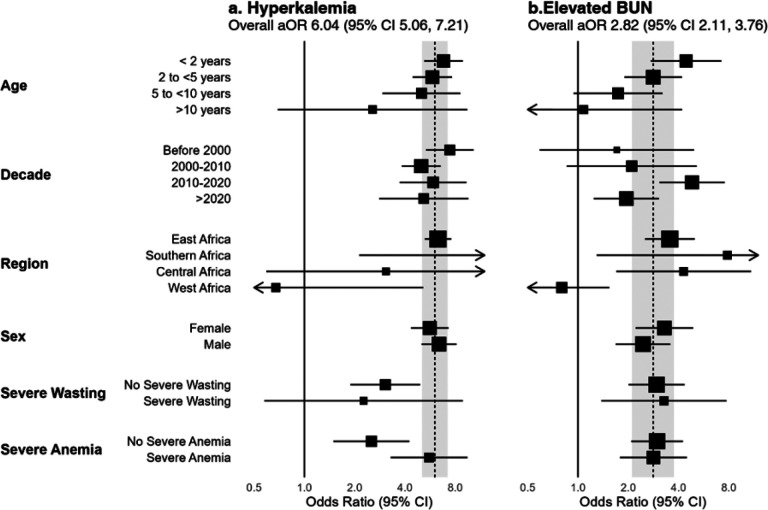
Relationship between hyperkalemia and elevated BUN on mortality. A separate mixed effect logistic regression model was fit for each sub-group, adjusting for age. The dashed line and shaded area represent the overall effect of hyperkalemia (a) or elevated BUN (b) on mortality (adjusted odds ratio [aOR] and 95% confidence interval [CI], adjusted for age. Values shown in Additional File 1, Tables S3 and S4. Two-stage analysis shown in Additional File 1, Figure S4

Complications of AKI include the accumulation of nitrogenous waste products (e.g., BUN) and electrolyte abnormalities, including hyperkalemia [34]. As secondary exposures, we evaluated the relationship between elevated BUN, hyperkalemia, and mortality. Hyperkalemia occurred in 7.6% of patients, and 29.3% of children had elevated BUN. Hyperkalemia rates were highest among children under 2 years (9%) and declined with age to 5% in children >10 years (p<0.0001), while elevated BUN was lowest in children under 2 years (19%) and increased across age groups to 45% in children >10 years (p<0.0001). There was evidence of heterogeneity across regions for both hyperkalemia and elevated BUN, with neither associated with mortality in studies from West Africa (Figure 5).

Overall, both hyperkalemia and elevated BUN were associated with mortality (pooled OR 6.04 [95% CI 5.06 to 7.21] and 2.82 [2.11 to 3.76], respectively).

### The effect of AKI complications hyperkalemia and elevated BUN on mortality

**Figure 6:**
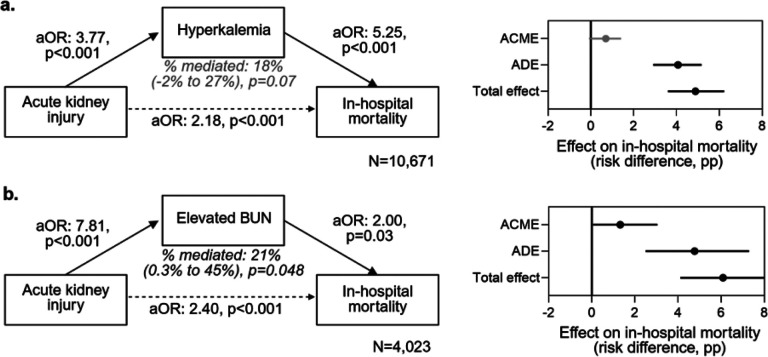
Causal mediation of the association between acute kidney injury (AKI) and in-hospital mortality by (a) hyperkalemia and (b) elevated BUN. Left panels show adjusted odds ratios (aOR) for the AKI-mediator association and mediator-mortality association; the dashed arrow depicts the direct AKI-mortality path for context. Models are adjusted for age, sex, decade, and region. Right panels display the average causal mediation effect (ACME), average direct effect (ADE), and total effect (ACME + ADE) on the risk-difference scale reported as percentage points (pp) with 95% confidence intervals. Black/solid denotes significant mediation (ACME p<0.05); grey denotes non-significant mediation. “% mediated” and its 95% CI are derived from the mediation model.

Mediation analysis was used to evaluate the relationship between AKI and elevated BUN or hyperkalemia on mortality. Among children with hyperkalemia, 83.2% had AKI, and among children with elevated BUN, 73.4% had AKI. In causal mediation models adjusted for age, sex, decade, and region, elevated BUN explained 21% of the association between AKI and in-hospital mortality (p=0.048). Hyperkalemia explained 18% of AKI-related mortality and did not reach statistical significance (p=0.07) (Figure 6).

## Discussion

This individual patient data meta-analysis of African children with *Plasmodium falciparum* malaria included data from 13,528 children collected over 33 years across eight countries. Using the KDIGO criteria, AKI occurred in 47% of children with severe malaria and is a robust and independent predictor of mortality (OR 2.87 [95% CI 2.28 to 3.61]). Subgroup analyses indicate that the high burden of AKI was consistent across time, by age, and associated with mortality across all regions in Africa.

In Africa, severe malaria cases and deaths are highest in children less than 2 years of age and are less frequent amongst older children and adults due to the acquisition of naturally acquired immunity [35, 36]. In this vulnerable population, the frequency of community-acquired AKI was 47%, with 67% of deaths occurring in the context of AKI. Previously, AKI was considered more common in older children and adults when applying a single creatinine threshold of 3mg/dL to define the disease [2]. However, when applying age-appropriate approaches to estimate baseline creatinine, the frequency of AKI was lowest in older children. The relationship between AKI and mortality across age groups emphasizes the need for increased education and awareness of AKI in young children, where AKI occurs at low creatinine thresholds compared to older children and adults. Proposed age-based thresholds to facilitate AKI recognition by age and across AKI stages are outlined in Table 1. These thresholds are based on simplified age-based thresholds derived from baseline creatinine estimates, rounded and grouped by age to facilitate bedside recognition of AKI. This approach had comparable performance to KDIGO-defined AKI and had minimal misclassification of severe (stage 2 or 3) AKI.

When evaluating AKI during an initial clinical encounter or in an epidemiological setting, baseline creatinine should be estimated, as most AKI in severe malaria is community-acquired[37]. Approaches to estimating baseline creatinine, validated in African pediatric cohorts, assume an estimated glomerular filtration rate (eGFR) of 120mL/min per 1.73m^2^ and back-calculate creatinine using age-based equations [32, 38]. One approach to mitigate the risk of over-diagnosing AKI due to imprecision in creatinine estimates involves applying a minimum creatinine threshold to define AKI [39, 40]. Thresholds of 0.5mg/dL have been recommended in high-resource settings[39] but may not be appropriate in low-resource settings where undernutrition is common. In this population, applying a minimum threshold of 0.5mg/dL results in a significant reduction in AKI frequency, particularly in children < 2 years. Applying a minimum threshold of 0.4mg/dL prevents over-estimation at low creatinine values, where assay precision is a concern, but does not impact AKI detection in children ≥ 2 once GFR has reached its stable value. It is important to note that in children with low baseline creatinine values (e.g., younger children, children with undernutrition), AKI may occur even when the creatinine is within normal reported laboratory ranges, and failure to identify AKI in the early stages when it is amenable to interventions can result in the progression of the disease and the need for dialysis.

Given the high frequency of AKI identified in this study, we suggest all children with severe malaria should be considered at high risk for the development of AKI, with simplified age-based thresholds for identifying AKI proposed. Clinicians caring for children with severe malaria should implement kidney-focused care by monitoring creatinine levels and urine output, avoiding nephrotoxic medications when possible, and paying careful attention to patient volume status. Fluid balance may be monitored by taking daily weights, using the first measurement as the anchor weight and the same scale for all measurements. While this approach has limitations in an intensive care setting, where children with AKI may be mechanically ventilated or require extracorporeal therapies, most severe malaria is managed on general wards, and taking daily weights should be a feasible approach to assess fluid balance and identify positive fluid balances that may denote fluid overload [41]. Maintaining vigilance for AKI-associated complications such as hyperkalemia, acidosis, and volume overload, and being prepared to manage them as they arise, are the cornerstones of kidney-centered care for children with malaria. The findings of differences in hyperkalemia and BUN-associated mortality in West Africa call for additional research to define the etiology of AKI-related complications across geographic contexts. However, with most participants from East Africa (89%) and 58% from Kenya, broader representation from the rest of Africa is urgently needed. Regularly dosed paracetamol for 72 hours in adults with severe falciparum malaria and severe knowlesi malaria with evidence of intravascular hemolysis showed evidence of being kidney protective in randomized controlled trials [42, 43]. The role of regular paracetamol as a kidney protective agent in children with severe falciparum malaria in Africa is the subject of ongoing clinical trials (NCT04251351, ISRCTN84974248).

Enhanced awareness of malaria as a risk factor for AKI should prompt enhanced screening for AKI complications and implementation of triage systems to guide resource allocation and transfer to higher-level facilities for advanced monitoring and dialysis, where available. Lower mortality rates in studies and centers where dialysis was available, even with limited availability, suggest that conservative measurement of AKI complications can reduce mortality independent of dialysis. Thus, increased awareness of AKI and education on how to recognize and manage AKI are critical to support AKI recovery, reduce mortality, and avert the need for dialysis [44]. For patients with worsening kidney dysfunction, early referral for organ support with dialysis should be considered. AKI-associated mortality was significantly lower when dialysis was available, even after adjusting for time, antimalarial therapy, and other clinical complications.

The overall mortality rate in children with malaria receiving dialysis was 18% — lower than previous estimates of 34% in African children receiving dialysis for any cause of AKI[45]— and consistent with reports of improved survival in severe malaria-associated AKI compared to other infectious causes of AKI [46]. Hemodialysis in children is complicated by issues with vascular access, limited pediatric supplies, and risks of hemodynamic instability, making peritoneal dialysis the preferred modality for younger children. Expanded access to peritoneal dialysis using improvised materials has the potential to avert deaths [47–49]. Late recognition and referral may contribute to increased mortality, particularly the high mortality among children receiving peritoneal dialysis, which is often implemented as a last resort. Therefore, there is an urgent need to improve access to acute dialysis as a life-saving therapy for children with severe malaria-associated AKI, with 6 to 14% of children with severe malaria having a dialysis indication[28].

While deaths in children with severe malaria-associated AKI may not be directly attributable to AKI, severe malaria complications, including electrolyte abnormalities and acidosis, will be exacerbated and more challenging to manage when kidney function is compromised. Mediation analysis showed that up to 21% of AKI-associated mortality was partially mediated through elevated BUN (> 20mg/dL). However, it is important to note that the BUN cut-off reflects prognostic thresholds identified in children with severe malaria [50] but is well below the threshold typically associated with uremic symptoms. In the context of severe malaria, where thrombocytopenia is common, uremia (BUN > 80mg/dL) may further impact platelet function, increasing both bleeding and thrombosis risk. It can contribute to cerebral edema and raise intracranial pressure in children with neurologic findings. Additionally, uremia may increase vascular permeability, leading to pulmonary edema and respiratory failure requiring advanced respiratory support. Further, AKI was associated with hyperkalemia, and hyperkalemia was associated with mortality. Hyperkalemia is more likely to occur in patients with severe malaria due to intracellular potassium shifts from intravascular hemolysis, metabolic acidosis, and decreased potassium excretion in AKI. Emergency interventions to reduce mortality from fatal cardiac dysrhythmias include stabilizing the cardiac membrane, moving potassium intracellularly, and correcting acidosis [34]. In non-emergent settings, loop diuretics can be used to increase potassium excretion in the urine, and cation exchange resins can increase bowel losses of potassium [34]. Overall, early recognition of AKI and its complications offers opportunities for clinical interventions to prevent AKI progression, avert the need for dialysis, and reduce AKI-related mortality.

This individual patient-level meta-analysis included a large sample size encompassing severe malaria hospitalizations over 33 years and representation from across Africa, demonstrating that AKI is a clear and consistent risk factor for mortality. We used a single creatinine measurement at enrollment to increase the number of eligible studies. The results remained consistent over time, independent of variations in creatinine assay methodology. While some studies had a moderate risk of bias, predominantly due to the selection of children with certain features of severe malaria (e.g., coma, dark urine), the findings were consistent across studies. Data largely reflect findings from East Africa, where malaria transmission is stable. We lack comprehensive data from Western Africa, a region characterized by seasonal malaria transmission, where a relatively high incidence of kidney risk variants (e.g., *APOL1*), which may influence susceptibility to AKI and affect kidney recovery [51, 52].

Overall, mortality is estimated at 7% among children hospitalized with severe malaria. Assuming 597,000 malaria deaths annually, we estimate there are over 8 million severe malaria cases each year. AKI occurs in roughly 40% of children and adults with severe malaria, which equates to more than 3 million AKI cases attributable to malaria every year. Although the International Society of Nephrology aims to eliminate preventable deaths from AKI by 2025 [53], we remain far from achieving this goal. Severe malaria-associated AKI is a preventable cause of death, and public health efforts to prevent infection through vector control programs, vaccination, and early and effective treatment of infection can avert substantial morbidity and mortality.

## Supplementary Material

Supplementary Files

This is a list of supplementary files associated with this preprint. Click to download.

- AdditionalFile1IPDAKIFiguresandTablessubmit.docx
- AdditionalFile2IPDAKIMethods.docx
- AdditionalFile3IPDAKIExplanationofNsfordifferentanalyses.docx
- Table2.docx

Table 2 is available in the Supplementary Files section

## Figures and Tables

**Table 1.** Proposed Serum Creatinine-associated AKI thresholds by age

Age in years	Estimated baseline		‘At Risk’ Stage 1 AKI		‘Severe AKI’ Stage 2		‘Severe AKI’ Stage 3	
	μmol/L	mg/dL	μmol/L	mg/dL	μmol/L	mg/dL	μmol/L	mg/dL
1	18	0.2	35	0.4	45	0.5	62	0.7
2 to 5	27	0.3	45	0.5	55	0.6	80	0.9
6 to 9	35	0.4	55	0.6	70	0.8	106	1.2
10 to 13	44	0.5	70	0.8	90	1.0	133	1.5
14 to 16	53	0.6	80	0.9	105	1.2	159	1.8

Serum creatinine thresholds obtained from Pottel age-based eGFR equation assuming a normal GFR of 120mL/min per 1.73m^2^. Values were rounded across age groups for the normal range and multiplied by 1.5 for Stage 1 and 2 for Stage 2+ AKI.

**Table 3.** Mortality by AKI definition and stage

	Total N	N (%) died	aOR (95% CI)
**KDIGO-defined AKI**			
No AKI	4398	152 (3.5)	Reference
Stage 1	3164	151 (4.8)	1.63 (1.28, 2.07)
Stage 2	3172	212 (6.7)	2.35 (1.86, 2.97)
Stage 3	2794	436 (15.0)	6.37 (5.10, 7.95)
**Operationalized age thresholds**			
No AKI	5660	204 (3.6)	Reference
Stage 1	1340	63 (4.6)	1.36 (1.00, 1.84)
Stage 2	3840	269 (7.0)	2.15 (1.76, 2.63)
Stage 3	2688	416 (15.5)	5.60 (4.59, 6.83)
**WHO definition**			
Renal impairment (creatinine>3mg/dL)	272	47 (17.3)	5.58 (3.86, 8.07)

Mixed-effect logistic regression model adjusted for age and assay type

## Data Availability

The datasets generated and/or analyzed during the current study are available in the Harvard Dataverse. https://doi.org/10.7910/DVN/EBQNQD.
